# Synthesis and Characterization of New Optically Active Poly (ethyl L-lysinamide)s and Poly (ethyl L-lysinimide)s

**DOI:** 10.4061/2010/910906

**Published:** 2010-07-13

**Authors:** Saeed Zahmatkesh, Mohammad Reza Vakili

**Affiliations:** ^1^Department of Science, Payame Noor University (PNU), Tehran 19569, Iran; ^2^Department of Chemistry, Islamic Azad University, Firouzabad, 74715-117 Fars, Iran

## Abstract

Ethyl L-lysine dihydrochloride was reacted with three different dianhydrides to yield the poly (ethyl L-lysinimide)s (**P** **I**_**1**−**3**_); it was also reacted with two different diacyl chlorides to yield the poly (ethyl L-lysinamide)s (**P** **A**_**4**-**5**_). The resulting polymers have inherent viscosities in the range of 0.15 to 0.42 dL g^−1^. These polymers are prepared from an inexpensive starting material and are optically active, potentially ion exchangeable, semicrystalline, thermally stable, and soluble in polar aprotic solvents such as DMF, DMSO, NMP, DMAc, and sulfuric acid. All of the above polymers were fully characterized by FT-IR and ^1^H NMR spectroscopy, elemental analysis, WAX diffraction, TGA, inherent viscosity measurement, and specific rotation.

## 1. Introduction

Polyamides, Polyimides, and their copolymers are certainly one of the most useful classes of high-performance polymers, which have found many applications in industries as discussed by Mittal [1] and Abade [2]. Aromatic polyimides are an important class of heterocyclic polymers with remarkable heat resistance and superior mechanical and electrical properties, and also durability as discussed by Banihashemi and Abdolmaleki [3], Ghosh and Mittal [4] and Wilson et al. [5]. Various efforts have been focused on the preparation of soluble and/or thermoplastic polyimides, while still maintaining the excellent thermal and mechanical properties. Typical approaches that have been employed to improve the processability of them include the incorporation of flexible links as discussed by Tamai et al. [6], bulky pendant or cardo groups as discussed by Hsiao and Li [7] and Müller and Ringsdorf [8], kinked or asymmetric structures as discussed by Li et al. [9], and spiro skeletons as discussed by Reddy et al. [10] into the polymer chain. These modifications lower the melting temperature and lead to soluble and amorphous polymers. In general, amorphous polymers have a lower softening temperature (*Tg*) and improved solubility with respect to their crystalline analogues. Some of the block copolymers composed of polyethers and polyamides have already been commercialized as thermoplastic elastomers as discussed by Legge et al. [11]. A number of synthetic routes for polyether-polyimide block copolymers have been known as discussed by Noshay and McGrath [12]. The synthesis and application of optically active polymers is a considerable topic, which has been paid more attention recently as discussed by Hajipour et al. [13]. Most of the natural polymers are optically active and have special chemical activities, such as catalytic properties that exist in genes, proteins, and enzymes. Some other applications are construction of chiral media for asymmetric synthesis, chiral stationary phases for resolution of enantiomers in chromatographic techniques as discussed by Akelah and Sherrington [14], Aglietto et al. [15], Yuki et al. [16], Okamoto and Yashima [17], and Soai and Niwa [18], chiral liquid crystals in ferroelectrics and nonlinear optical devices as discussed by Wulff [19] and Fontanille and Guyot [20]. These synthetic polymers based on optically pure amino acids can induce crystallinity with their ability to form higher ordered structures that exhibit enhanced solubility characteristics as discussed by Birchall et al. [21]. These properties have caused them to be good candidate for drug delivery systems, biodegradable macromolecules, biomaterials, and also as chiral purification media as discussed by Mallakpour et al. [22]. So, more considerations to improve different synthetic procedures of optically active polymers exist. Recently, we have synthesized optically active polymers by different methods as discussed by Mallakpour et al. [23, 24] and Hajipour et al. [25]. L-lysine with good functionalities has been used to prepare some polytartaramides as discussed by Bou and Muñoz-Guerra [26] and Majό et al. [27]. In this research, we report the synthesis and characterization of some **PA**s and **PI**s from an inexpensive starting material through polycondensation in refluxing DMF. These polymers showed good optical activity (−28.12° to −48.56°) and because of the presence of pendent ester moiety they can potentially be ion exchangeable as discussed by Müller and Ringsdorf [8]. In **PI_2_** because of the presence of benzophenone moiety, this polymer can potentially be photolabile. The photolabile polymers can potentially be used as affinity columns for protein purification as discussed by Guo et al. [28]. The outstanding characteristics of these polymers include thermal stability, good solubility, optical activity, semicrystallinity, potentially being photolabile and ion exchangeable.

## 2. Material and Methods

The dianhydrides (Merck) were recrystallized from acetic anhydride. The other chemicals (Merck) were used as received. ^1^H NMR spectra were recorded on 300 MHz (Bruker Avance) instrument, using DMSO-d_6_ as solvent and tetramethylsilane as shift reference (tube diameter, 5 mm). IR spectra were recorded on a Shimadzu FT-IR-680 instrument, using KBr pellets. Specific rotations were measured by a JASCO P-1030 Polarimeter in DMF s solvent. UV spectra were recorded on a JASCO V-570 instrument in DMF solvent. Thermogravimetric analyses (TGA) were recorded on a Mettler TGA-50 with heating rate of 10°C min^−1^ under air atmosphere. Inherent viscosities of polymers were measured by a standard procedure using a KPG Cannon Fenske routine viscometer at 25°C using DMF as solvent. Melting points were measured in open capillaries with a Gallenkamp instrument. Elemental analyses were preformed in a Heraeus CHNS-RAPID instrument.

### 2.1. Monomer Synthesis

#### 2.1.1. Synthesis of Ethyl L-Lysine Dihydrochloride (as discussed by Bou et al. [29])

In a 50 mL round-bottomed flask equipped with a reflux condenser and a stirring bar, 8 mL of thionyl chloride was added dropwise to the stirring absolute ethanol (2.5 mL) at −10°C. L-lysine hydrochloride (7.3 g, 0.04 mol) was added to the mixture and refluxed for 6 hours. The solvent was evaporated under reduced pressure and the residue was washed with diethyl ether for three times. Yield: 87%; m.p.: 136-137°C; IR (cm^−1^): 3421, 3350–2514, 2019, 1740, 1603, 1583, 1501, 1217, 851, 740; ^1^H-NMR (D_2_O, ppm): 1.07 (3H), 1.29 (2H), 1.49 (2H), 1.76 (2H), 2.78 (2H), 3.91 (1H), 4.08 (2H); Elemental analysis for C_8_H_18_N_2_O_2_·2HCl, Calculated: C (38.87%), H (8.16%), N (11.33%), Found: C (38.62%), H (8.31%), N (11.40%).

#### 2.1.2. Poly (ethyl L-lysinimide)s Synthesis, General Procedure

For the general procedure in a 25 mL round-bottomed flask equipped with a reflux condenser and a stirring bar, a mixture of dianhydride (0.001 mol), ethyl L-lysine dihydrochloride (0.001 mol), Et_3_N (0.002 mol), and DMF (5 mL) were placed. The mixture was stirred at r.t. for 2 hours and then at refluxing temperature for 5 hours. The mixture was poured dropwise into 15 mL of H_2_O. The white precipitate was filtered off, washed with water, and dried under vacuum condition.

**PI_1_ (using pyromellitic dianhydride)**

Yield: 75%; *η*_inh_′ (dL g^−1^) = 0.42; [*α*]_*D*_^25^ = −30.68°; UV (*λ*_max⁡_): 263; IR (cm^−1^): 2980–2894, 2858, 1774–1680, 1495, 1458–1386, 1244, 1120, 1023, 719, 496; ^1^H NMR (DMSO-d_6_, ppm): 1.26 (2H), 1.41 (3H), 1.76–1.83 (2H), 2.33 (2H), 3.74 (2H), 4.23 (1H), 4.87 (2H), 8.06–8.29 (2H). Elemental analysis for C_18_H_16_N_2_O_5_: Calculated: C (63.52%), H (4.73%), N (8.23%), Found: C (63.42%), H (4.80%), N (8.11%). Wide-angle X-ray diffraction patterns of this polymer in the region of 2*θ* = 5–70° at room temperature indicate 20–30% of crystallinity.

**PI_2_(using 3,3′,4,4′-benzophenone tetracarboxylic-3,3′,4,4′-dianhydride)**

Yield: 80%; *η*_inh_′ (dL g^−1^) = 0.15; [*α*]_*D*_^25^ = −48.56°; UV (*λ*_max⁡_): 265, 290; IR (cm^−1^): 2938, 1776–1715, 1669, 1619, 1441, 1385, 1294, 1246, 1184, 1157, 1096, 1024, 859, 726; ^1^H NMR (DMSO-d_6_, ppm): 1.26 (2H), 1.44 (3H), 1.75–1.82 (2H), 2.34 (2H), 3.73 (2H), 4.24 (1H), 4.87 (2H), 7.99–8.23 (6H). Elemental analysis for C_25_H_20_N_2_O_6_: Calculated: C (67.55%), H (4.54%), N (6.30%), Found: C (67.46%), H (4.77%), N (6.16%).

**PI_3_(using 4,4′-(hexaflouroisopropylidene) diphthalic anhydride)**

Yield: 70%; *η*_inh_′ (dL g^−1^) = 0.18; [*α*]_*D*_^25^ = −28.26°; UV (*λ*_max⁡_): 280; IR (cm^−1^): 2941, 1779–1719, 1442, 1385, 1297, 1255, 1210, 1140, 1105, 722; ^1^H NMR (DMSO-d_6_, ppm): 1.05–1.12 (2H), 1.22 (3H), 1.53–1.58 (2H), 2.11 (2H), 3.49 (2H), 4.01 (1H), 4.65 (2H), 7.60–7.99 (6H). Elemental analysis for C_27_H_20_N_2_O_5_F_6_: Calculated: C (57.24%), H (3.56%), N (4.95%), Found C (57.10%), H (3.64%), N (4.86%).

#### 2.1.3. Poly (ethyl L-lysinamide)s Synthesis, General Procedure

In a 25 mL round-bottomed flask equipped with a reflux condenser and a stirring bar, a mixture of aromatic acid dichloride (0.001 mol), ethyl L-lysine dihydrochloride (0.001 mol), Et_3_N (0.004 mol) and DMF (5 mL) were placed. The mixture was stirred at r.t. for 10 hours. The mixture was poured dropwise into 15 mL of H_2_O. The white precipitate was filtered off, washed with water, and dried under vacuum conditions.

**PA_4_(using terephthaloyl dichloride)**

Yield: 65%; *η*_inh_′ (dL g^−1^) = 0.31; [*α*]_*D*_^25^ = −28.12°; UV (*λ*_max⁡_): 265; IR (cm^−1^): 3423, 2963, 1727–1615, 1505, 1439, 1410, 1276, 1197, 1118, 1018, 727; ^1^H NMR (DMSO-d_6_, ppm): 1.12 (2H), 1.23–1.26 (3H), 1.32 (2H), 1.38–1.60 (2H), 1.82 (2H), 2.96 (2H), 3.29 (1H), 4.30 (1H), 4.47 (2H), 7.65–8.16 (4H), 11.42 (1H). Elemental analysis for C_16_H_20_N_2_O_3_: Calculated C (66.65%), H (6.99%), N (9.71%), Found C (66.54%), H (7.07%), N (9.66%).

**PA_5_(using isophthaloyl dichloride)**

Yield: 60%; *η*_inh_′ (dL g^−1^) = 0.17; [*α*]_*D*_^25^ = −30.22°; UV (*λ*_max⁡_): 265; IR (cm^−1^): 3422, 2962, 1722–1685, 1431, 1312, 1288, 1251, 1131–1077, 724; ^1^H NMR (DMSO-d_6_, ppm): 1.12 (2H), 1.23–1.26 (3H), 1.39 (2H), 1.40–1.79 (2H), 2.97 (2H), 3.25 (1H), 4.42 (2H), 7.64–8.56 (5H). Elemental analysis for C_16_H_20_N_2_O_3_: Calculated C (66.65%), H (6.99%), N (9.71%), Found C (66.48%), H (7.10%), N (9.62%).

## 3. Results and Discussion

Ethyl L-lysine dihydrochloride was prepared with the reaction of a mixture of EtOH and thionyl chloride with L-lysine hydrochloride. L-lysine hydrochloride was added to the mixture dropwise at −10°C and then refluxed for 6 hours. The dark solid was washed three times with diethyl ether to leave a bright white solid (87%). FT-IR spectroscopy shows a strong and broad peak at 3350–2514 cm^−1^ corresponding to the Amonium N–H stretchings and a strong peak at 1740 cm^−1^ corresponding to the C=O stretchinng of ester moiety. ^1^H-NMR (D_2_O, ppm) spectroscopy shows the corresponding peaks such as 3.91 (1H) due to the chiral center and 1.07 (3H) and 2.78 (2H) peaks due to the ethyl moiety.

**Scheme 1:**
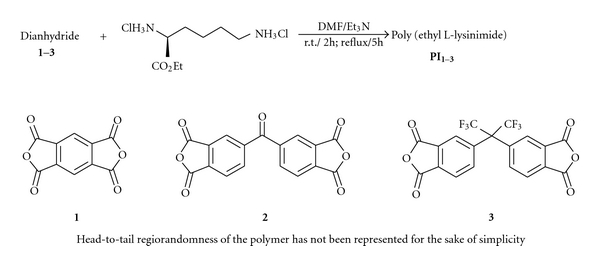
Poly (ester-imide) synthesis.

**Scheme 2:**
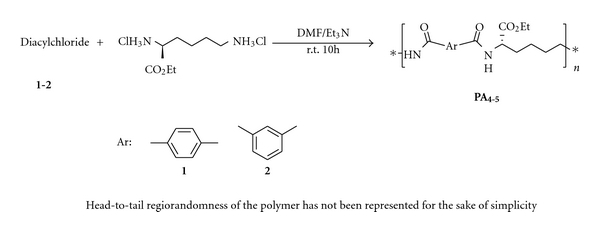
Poly (amide-imide) synthesis.

Solution polymerization in DMF in the presence of Et_3_N was applied to prepare the polymers from ethyl L-lysine dihydrochloride and the other corresponding monomers (Schemes 1 and 2). **PA**s were prepared at r.t. but in preparing the **PI**s the mixture was refluxed to turn the amic acid groups to imide. Et_3_N was used to release the amino group of the L-lysine derivative and also to scavenge the released HCl in amidation. We also found that using an ionic liquid (1-methyl-3-propyl imidazolium bromide) as the solvent of polymerization can cause some difficulties in purification, so it is an unsuitable polymerization media.

**Figure 1:**
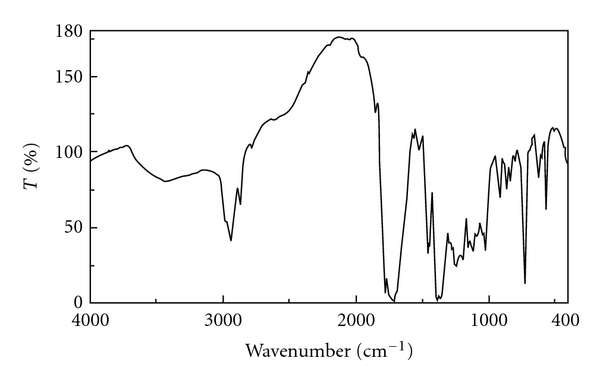
IR spectrum of **PI_1_**.

**Figure 2:**
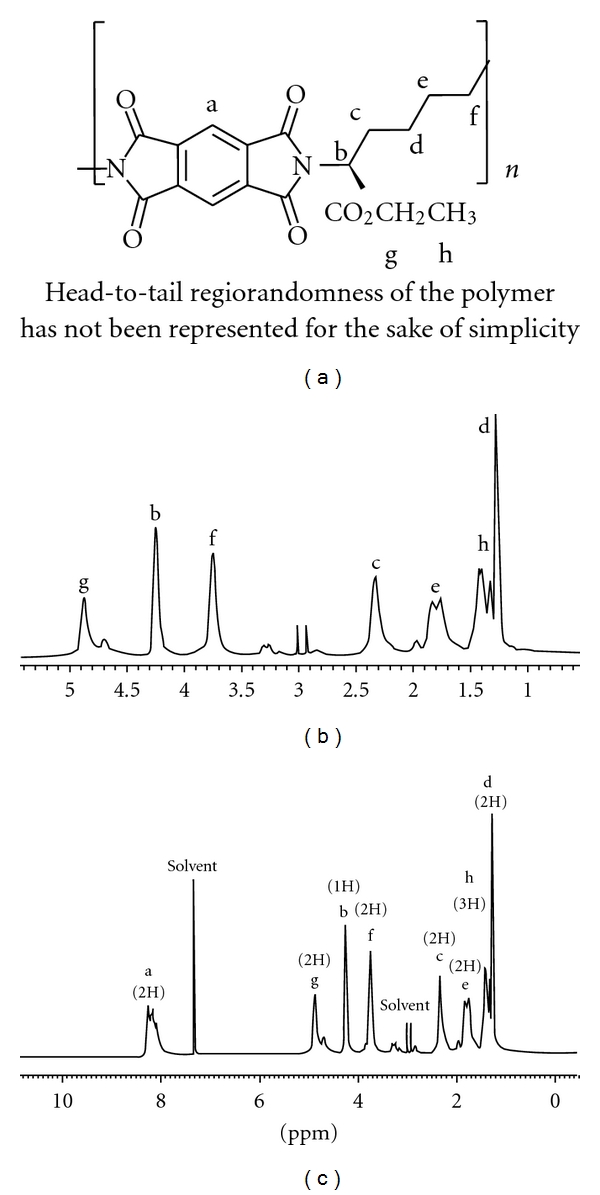
^1^H NMR spectrum of very probable atactic **PI_1_**.

**Figure 3:**
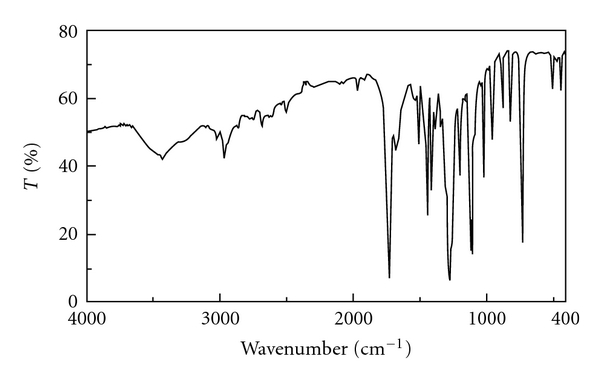
IR spectrum of **PA_4_**.

**Figure 4:**
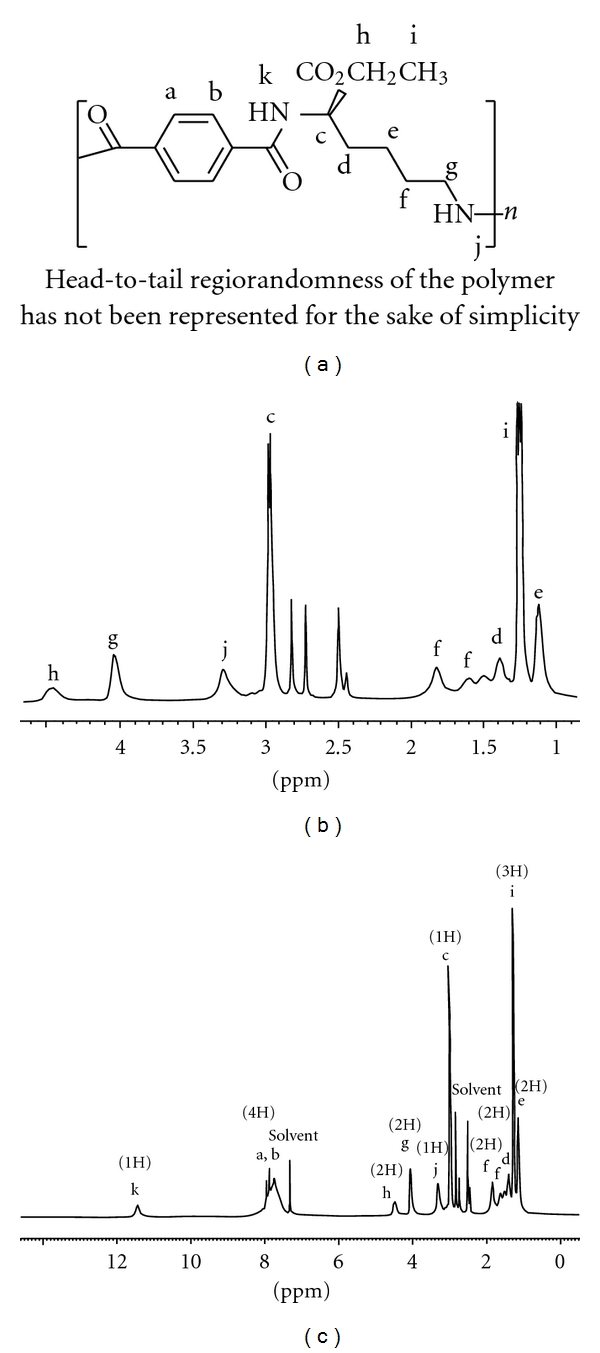
^1^H NMR spectrum of very probable atactic **PA_4_**.

All of the very probable atactic polymers were obtained from an inexpensive starting material in quantitative yields with moderate inherent viscosities (0.15–0.42 dL g^−1^) and optical rotation (−28.12° to −48.56°). As there is no obvious regioselectivity between alpha and epsilon amino groups of the lysine ester during the polymerisation step then random orientation of lysine moieties along the polymer backbone can be predicted and the concept of “tacticity" cannot be addressed in this research. Head-to-tail regiorandomness may likely affect some physical properties of the polymers such as crystallinity. The formation of **PI**s was confirmed by IR and ^1^H NMR spectroscopy and elemental analysis. As an example, the IR of **PI_1_** showed the C=O asymmetric stretching of imide group, the C=O symmetric stretching of imide and ester groups at 1774–1680 cm^−1^, C–N stretching at around 1386 cm^−1^, and C–O stretching at 1120 cm^−1^. All of these **PI**s exhibited strong absorption at around 1380 and 720 cm^−1^, which shows the presence of the heterocyclic imide groups (Figure 1). As an example the ^1^H NMR spectrum of **PI_1_** is presented in Figure 2 which shows peaks that confirmed its chemical structure (^1^H NMR (DMSO-d_6_, ppm): 1.26 (2H), 1.41 (3H), 1.76–1.83 (2H), 2.33 (2H), 3.74 (2H), 4.23 (1H), 4.87 (2H), 8.06–8.29 (2H)). The formation of **PA**s was confirmed by IR and ^1^H NMR spectroscopy and elemental analysis. As an example, the IR of **PA_4_** showed the N–H stretching of amide group at 3423 cm^−1^, the C=O stretching of amide and ester and also the N–H bending of amide groups at 1727–1615 cm^−1^ and the C–O stretching at 1118 cm^−1^ (Figure 3). As an example the ^1^H NMR spectrum of **PA_4_** is presented which shows peaks that confirmed its chemical structure (Figure 4). The elemental analyses results are also in good agreement with calculated/expected percentages of carbon, hydrogen, and nitrogen contents in the polymer-repeating units. The color of these polymers was white. The resulting homogenous glassy compound films were isolated by adding methanol/H_2_O (80 : 20) and triturating, followed by filtration. It was washed several times with methanol and vacuum dried. Transparent, flexile, and tough films of these polymers were obtained which showed good mechanical strength of the films and consequently high molecular weight. Wide-angle X-ray diffraction patterns of **PI_1_** in the region of 2*θ* = 5–70° at room temperature indicate 20–30% of crystallinity. TGA technique shows moderate to good thermal stability for these polymers (Table 1). For example the TGA/DTG spectrum of **PI_1_** presents *T*_5_% and *T*_10_% at around 240°C and 310°C, respectively. These polymers can be partially hydrolyzed to present the pendent carboxylic acid groups (ion-exchangeable polymers). **PA_5_** can potentially be photolabile and be used to prepare an affinity column. It is because of the presence of benzophenone moiety. One of the major objectives of this work is to study the solubility and the versatility of these polymers by incorporating the soft segment in the polymer backbone. The solubility of these polymers was tested qualitatively in various solvents and the results are summarized in Table 2.

## 4. Conclusions

Five new chiral polyamides and polyimides incorporating ethyl L-lysine ester have been synthesized from an inexpensive starting material by usual solution polycondensation method. These polymers are very soluble, optically active, potentially ion exchangeable and semicrystalline. The resulting polymers are identified spectroscopic methods such as FT-IR, UV-Vis and ^1^H NMR spectroscopy and elemental analysis. The polymers are characterized by yield of reaction, inherent viscosity, WAX diffraction, and specific rotation.

## Figures and Tables

**Figure 5:**
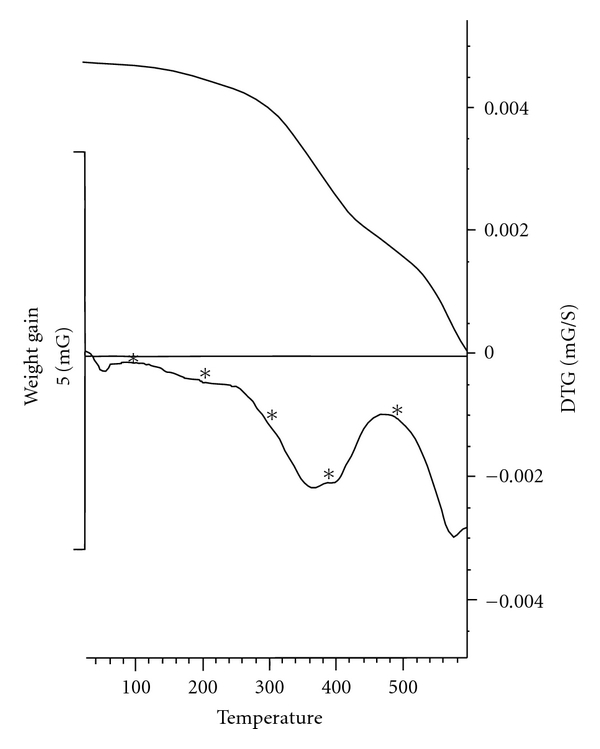
TGA/DTG spectrum of **PI_1_**.

**Table 1:** Thermal behavior of polymers.

Polymer	Decomposition temperature (°C) *T*_5%_^a^	Decomposition temperature (°C) *T*_10%_^b^	Char yield (%)^c^
**PI_1_**	240	310	5.2
**PI_2_**	225	300	6.4
**PI_3_**	230	335	7.0
**PA_4_**	280	350	3.4
**PA_5_**	265	345	2.5

^a^Temperature at which 5% weight loss was recorded by TGA at a heating rate of 10°C/min under air atmosphere. ^b^Temperature at which 10% weight loss was recorded by TGA at a heating rate of 10°C/min under air atmosphere. ^c^Percentage weight of material left after TGA analysis at maximum temperature 600°C under air atmosphere

**Table 2:** Solubility of polymers^a^.

Solvents	**PI_1_**	**PI_2_**	**PI_3_**	**PA_4_**	**PA_5_**
NMP	+	+	+	+	+
DMSO	+	+	+	+	+
DMAc	+	+	+	+	+
DMF	+	+	+	+	+
H_2_SO_4_	+	+	+	+	+
CH_2_Cl_2_	−	−	−	−	−
CHCl_3_	−	−	−	−	−
EtOH	−	−	−	−	−
MeOH	−	−	−	−	−
H_2_O	−	−	−	−	−

^a^Concentration: 5 mg mL^−1^: +, soluble at room temperature; −, insoluble.
